# *Citrus sinensis* zest-mediated silver nanoparticles for rapid dye removal and antimicrobial properties

**DOI:** 10.1039/d6ra02221a

**Published:** 2026-07-02

**Authors:** Mithun Rani Nath, Md Ruhul Amin Foisal, Imana Shahrin Tania, Abu Bin Imran

**Affiliations:** a Department of Chemistry, Bangladesh University of Engineering and Technology Dhaka-1000 Bangladesh mithun.acce@nstu.edu.bd abimran@chem.buet.ac.bd; b Department of Applied Chemistry and Chemical Engineering, Noakhali Science and Technology University Noakhali-3814 Bangladesh; c Department of Wet Process Engineering, Bangladesh University of Textiles (BUTEX) Dhaka-1208 Bangladesh

## Abstract

Growing interest in sustainable and renewable nanotechnology has accelerated the development of green synthesis strategies that are both environmentally friendly and economically viable. In this study, we report a simple, low-cost, and eco-conscious approach for synthesizing silver nanoparticles (AgNPs) using an aqueous zest extract of *Citrus sinensis* (*Cs*), which naturally serves as both the reducing and stabilizing agent. The phytochemicals present in the extract—including ascorbic acid, phenolic acids, flavonoids, terpenoids, and polysaccharides—effectively reduce Ag^+^ ions to stable AgNPs. The formation of AgNPs was confirmed through UV–Vis spectroscopy, revealing a characteristic surface plasmon resonance peak at 420 nm. FESEM imaging demonstrated predominantly spherical nanoparticles with uniform crystallite morphology and rough surface features, while TEM analysis showed an average particle size of approximately 15 ± 5.3 nm. XRD patterns confirmed the polycrystalline nature of the particles with diverse *d*-spacings, and EDX analysis indicated high purity (93.91% Ag) of the synthesized material. Compared to previously reported green synthesized nanoparticle systems, the biosynthesized AgNPs displayed excellent catalytic activity, achieving dye removal efficiencies of 98.93% (within 18 min) for methyl orange (MO) and 92.15% (within 30 min) for methylene blue (MB) under mild conditions, highlighting the advantage of waste-derived plant extract-mediated synthesis. Moreover, they exhibited strong antibacterial activity, showing notable inhibition against Gram-negative bacteria (*E. coli*,*Pseudomonas* spp.,*K. pneumoniae*, and*Salmonella* spp.) compared with Gram-positive strains (*S. aureus* and*B. cereus*), as assessed by inhibition zone measurements.

## Abstract 2

Growing interest in sustainable and renewable nanotechnology has accelerated the development of green synthesis strategies that are both environmentally friendly and economically viable.

## Graphical abstract

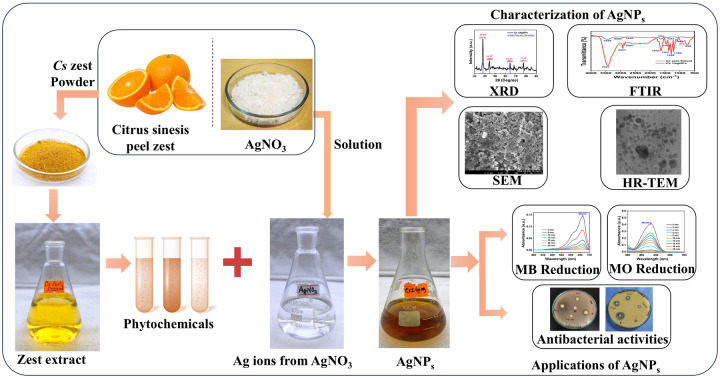

## 1 Introduction

Over the past several decades, nanotechnology has received growing attention for its ability to address critical water-related challenges, particularly those involving toxic organic dyes, heavy metals, antibiotics, and microbial contaminants. Nanoscience explores the unique physicochemical behaviors that emerge at the nanoscale, enabling the development of advanced materials, devices, and systems with novel functionalities.^1^ These particles have become highly valuable due to their extremely small size, large surface area-to-volume ratio, and distinctive chemical, physical, and biological characteristics, which differ substantially from their bulk counterparts.^2^ Owing to their enhanced catalytic, photocatalytic, electro-optical, and antimicrobial properties, nanomaterials have been widely investigated as promising tools for technological and environmental applications.^3^

Precious metals, such as gold, platinum, copper, zinc, and silver, are renowned for their chemical stability, distinctive optical properties, and diverse applications in catalysis, electronics, and medicine. Of these, AgNPs are preferred due to their strong antibacterial activity, chemical stability, catalytic activity, and ease of nanoparticle production.^4^ AgNPs demonstrate potent antimicrobial action against both Gram-positive and Gram-negative bacteria, primarily through interactions with cell membranes and disruption of essential cellular processes.^5^ They are also used in environmental applications, such as degrading toxic dyes and pollutants in wastewater due to their strong redox potential and surface activity.^6,7^

Although AgNPs have significant potential in nanoscience, biomedicine and environmental remediation, further research is needed to comprehend their biological interactions and harmful effects.^8^ Synthesis of AgNPs through traditional physical (*e.g.*, laser ablation, sputtering) or chemical (*e.g.*, vapor deposition, precipitation, condensation, pyrolysis) pathways using powerful reducing agents represents a significant hazard both from the production process perspective and for the environment.^9,10^ Additionally, these processes are typically energy-intensive, produce toxic byproducts, and require costly equipment^4^ Furthermore, controlling the stability and uniformity of nanoparticles from conventional routes is still a technical barrier.^11^

Consequently, green synthesis methods have emerged as an eco-friendly alternative. Biological routes utilizing microorganisms, fungi, bacteria, or plant extracts offer safer and more sustainable approaches to nanoparticle production.^12,13^ Compared to microbial synthesis routes, which are often time-consuming and require sterile conditions, plant-based synthesis is rapid and straightforward.^14^ It is therefore considered an economical and environmentally responsible strategy,^15^ as plant and fruit extracts are rich in abundant phytochemicals, which are responsible for the green synthesis of nanoparticles. They contain metabolites such as polyphenols, flavonoids, alkaloids, terpenoids, plant enzymes and phenolic compounds that naturally act as reducing agents and donate electrons to reduce metal ions during nanoparticle formation.^16,17^ While proteins, polysaccharides, and specific secondary phytochemicals serve as stabilizing or capping agents that bind to the nanoparticle surface, reducing aggregation and enhancing stabilization in the presence of metal ions.^18,19^

Numerous studies have confirmed that plant extracts can successfully produce stable and functional nanoparticles.^19–21^ Furthermore, the type and concentration of biomolecules are essential in influencing the dimension, structure, composition, functionality, and surface features of the nanoparticles produced.^22^ In particular, the applications of AgNPs in catalysis and biomedical fields depend significantly on their size and shape.^23,24^ Dong *et al.* discovered that a smaller particle size improves the antibacterial effectiveness of AgNPs, which were only tested on *Vibrio Natriegens*, hence limiting the applicability of the results to other bacterial species or conditions.^25^ Moreover, size-regulated AgNPs serve as a significant redox catalyst with a unique electron-transfer pathway, assisting in the decomposition of non-biodegradable organic pollutants in industrial wastewater. This study is hampered by the inadequate clarification of the AgNPs reduction mechanism and a restricted exploration of possible applications beyond those examined.^23,24^ In biosynthesized methods, different plant compounds are used as reducing and stabilizing agents for catalytic degradation and biomedical applications^26^^.^ Meanwhile, processing of food peel, zest, kernel, or pericarp waste to make AgNPs is a safe, long-lasting, and environmentally beneficial method compared to traditional methods.^8,27^

In this study, we sought to develop a green and optimized synthesis protocol for AgNPs production using the aqueous zest extract of Cs, commonly known as sweet orange or malta, an abundant agricultural waste resource. XRD, SEM, TEM, and FTIR, were employed to characterize the morphology, crystallinity, and structural properties of the synthesized AgNPs. The study systematically optimized Ag^+^ precursor concentrations (0.5–2.0 mM) and evaluated the catalytic performance of the optimized AgNPs for removing MB and MO dyes in the presence of NaBH_4_ as a reducing agent. Additionally, the biosynthesized AgNPs were examined for antibacterial efficacy against four Gram-negative and two Gram-positive bacterial strains. Beyond providing a simple synthesis route, the AgNPs produced exhibit excellent catalytic efficiency and strong antimicrobial activity.

## 2 Experimental

### 2.1 Materials

The chemicals used in this study were of analytical grade and were procured from commercial suppliers, requiring no additional purification. Silver nitrate (≥99.9%, analytical grade, Merck-KgaA, Germany) was used as the silver precursor, and sodium borohydride (99.9%) was obtained from Sigma-Aldrich, Germany. Fresh *Cs* fruit, commonly known as malta, was purchased from a nearby marketplace in Noakhali, Bangladesh, and subsequently stored for future usage. Deionized (DI) water was used consistently throughout the experiments. Methyl orange (MO) (85% dye content, ACS reagent) and methylene blue hydrate (MB) (≥95%, ACS reagent) were purchased from Merck, Germany. The BCSIR, Chattogram, Bangladesh, provided cultured bacteria. The glassware was cleaned with freshly distilled water, then dried in the oven before use. The antibacterial properties of AgNPs were investigated against four Gram-negative clinical pathogens: *Escherichia coli*,*Pseudomonas species*,*Klebsiella pneumoniae*, and*Salmonella species and* two Gram-positive pathogens, *Staphylococcus aureus* ATCC 25923 and Bacillus cereus. Tetracycline (30 µg), a broad-spectrum antibiotic, was obtained from Sigma-Aldrich (Merck, Germany) and used as a positive control in the antibacterial assay. Muller-Hinton agar (MHA) was supplied by Difco Laboratories, Detroit, USA.

### 2.2 Preparation of *Cs* fruit peel zest extract

Collected *Cs* fruits were cleaned with tap water two to three times to eliminate any dirt. Then washed with DI water, and the peel zest was chopped into tiny fragments and dried in the sunlight. Following drying, the zest was crushed into a fine powder and kept in sampling vials. The dried *Cs* zest powder was then added to a borosilicate glass flask, which contained a M : L ratio of 1 : 10. The solution was stirred at 80 °C for 2 hours. Finally, the yellowish-orange extract was cooled naturally, and the supernatant was filtered through Whatman No. 1 filter paper (pore size 11 micrometer, Fisher Scientific, USA). The resultant extract was kept in the refrigerator at 4 °C for future use.

#### 2.2.1 Synthesis of AgNPs

**Scheme 1:**
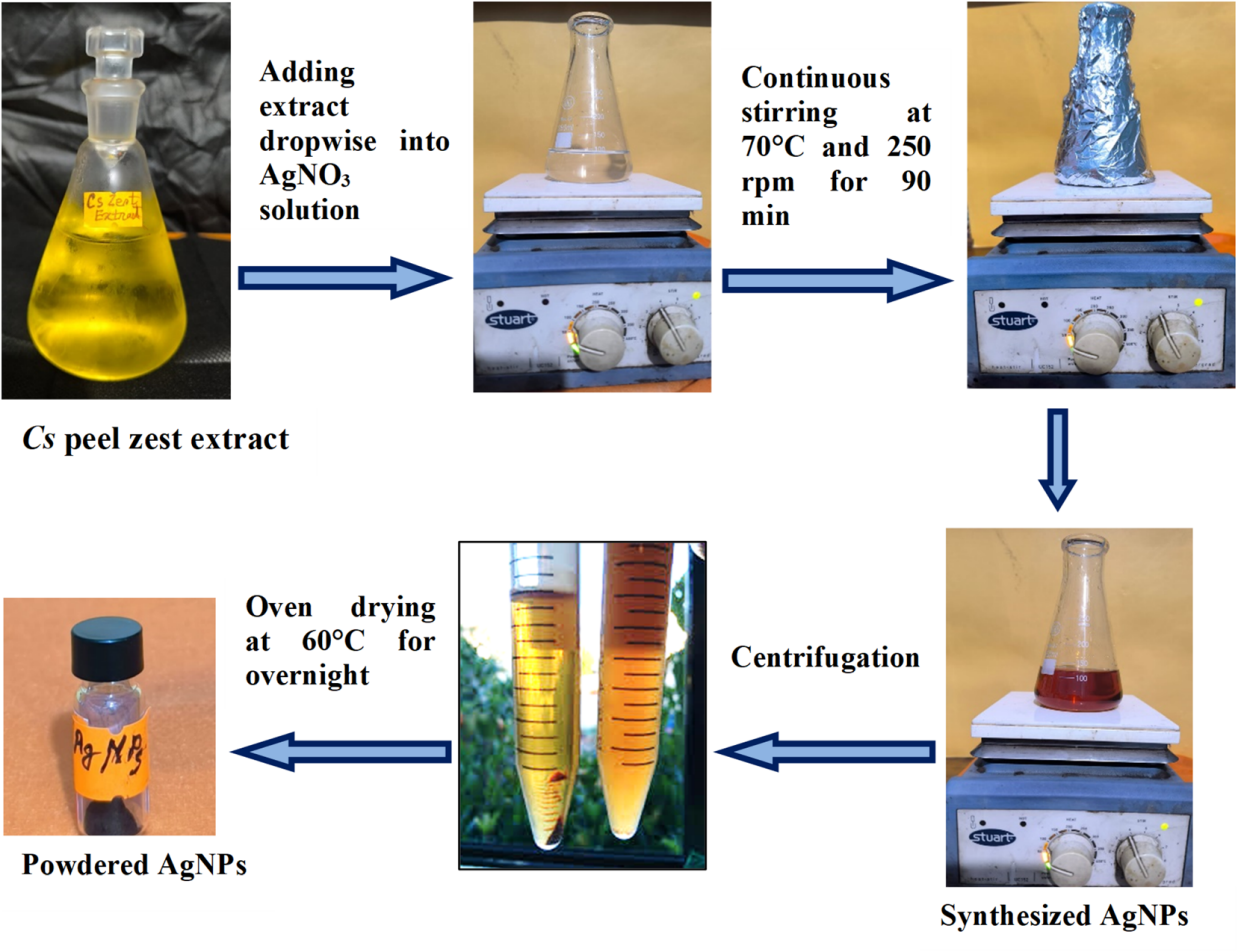
Experimental strategy to prepare AgNPs from *Cs* peel zest extract.

The biogenic synthesis of AgNPs is conducted in accordance with the literature,^28,29^ with minor modifications to the synthesis temperature, stirring time, and speed as described in the literature.^30^ Following this, 10 mL of the as-prepared Cs zest extract is added dropwise to 90 mL of a freshly prepared 1 mM solution of AgNO_3_. The solution was then stirred continuously for 90 minutes at 250 rpm and 70 °C using a magnetic stirrer to ensure complete nucleation and stability of AgNPs. The initial reduction of silver ions (Ag^+^) to AgNPs (Ag0) was verified by the observed color transition from a pale-yellow to a colloidal brown coloration. The prepared colloidal suspension was maintained at room temperature and allowed to stand undisturbed for 24 hours in the dark to ensure complete bio reduction. The purification of bio-generated nanoparticles was performed by centrifugation at 7000 rpm for 15 minutes. The precipitate was subsequently washed twice with deionized water and once more with ethanol. After washing, the AgNPs sample was dried overnight at 60 °C, then ground before storage at room temperature. The overall synthesis process is shown in Scheme 1. The procedure was separately performed for 0.5 mM, 1 mM, and 2 mM AgNO_3_ precursors, resulting in *Cs*0.5AgNPs, *Cs*1AgNPs, and *Cs*2AgNPs (*Cs* stands for *Cs*, 0.5–2 stands for millimolar concentration of AgNO_3_, and AgNPs).

### 2.3 Characterization of AgNPs

A double beam UV-visible spectrophotometer (Shimadzu, UV-1800, Japan) was used to confirm the synthesis of AgNPs. This characterization also allowed observation of the surface plasmon resonance (SPR) effect in the 350–700 nm range for AgNPs. Fourier Transform Infrared Spectra (Jasco, FTIR-6300, Japan) were recorded over the range 400–4000 cm^−1^ to compare the surface functional groups on the Cs zest extract and on the as-prepared AgNPs. An X-ray diffractometer (D8 Advance, Bruker, Germany) with Cu kα radiation (*λ* = 1.5406 Å) at a current of 40 mA and a voltage of 40 kV was used to get powdered X-ray diffraction patterns. The scan was performed over a 2*θ* range of 20° to 80°. XRD analysis was employed to determine the crystalline domain and the mean crystallite size using full-width at half-maximum (FWHM) data. Using the Debye–Scherrer equation (eqn (1)), we figured out the size of the AgNPs' crystallites by measuring the width of the Bragg reflection.^31^1
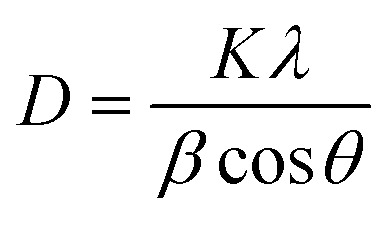
In this equation, *D* is the mean crystallite size, *λ* is the X-ray radiation wavelength (0.15406 nm), *β* is the FWHM (in radians), and *θ* is the Bragg reflection angle.

To analyze the surface morphology, particle size, and orientation of the prepared AgNPs, the field emission scanning electron microscope (FESEM) (JEOL, JSM-7600F, Japan) was used. ImageJ was used to determine the average size of the synthesized nanoparticles. The elemental composition of AgNPs was also measured using energy dispersive X-ray spectroscopy (EDX) at an acceleration voltage of 5 kV and 12 µA emission current. The HRTEM, JEOL-2100 equipment was also used to observe the clear morphology and size of the produced nanoparticles, as shown in TEM images at an accelerated voltage of 200 kV.

### 2.4 Catalytic reduction experiments

The catalytic degradation of two model pollutants, MB and MO, was investigated using biosynthesized AgNPs as catalysts in the presence of NaBH_4_. While the real wastewater system is complex, they are used as standard pollutants owing to their well-defined chemical structure and their strong characteristic absorption in the visible region, and ease of monitoring degradation kinetics prior to applying these methods to more intricate wastewater matrices.^32^ 20 mL of 5 mg L^−1^ dye solutions (MB, MO) were placed into a 50 mL conical flask, after which 100 µL of 0.01 M NaBH_4_ solution was added to each flask. Subsequently, 20 µL of suspensions containing various amounts of AgNPs (0.5 mM, 1 mM, 2 mM) was added to the mixture by stirring. The degradation of both pollutants was confirmed by discoloration.^33^ The concentrations (5 to 25 mg L^−1^) of dye solutions (MB and MO) were measured at various time points using a UV-Vis spectrophotometer at 664 nm (MB) and 464 nm (MO). The pH of the dye solutions was kept at neutral levels throughout the experiments.^33^

Beer–Lambert law has been used to describe a linear relationship between absorbance and reduction during the catalyst-mediated breakdown of pollutants.^34,35^

The catalytic reduction percentage (CR%) of both pollutants and the catalytic kinetics relationship between absorbance and reduction were determined using the pseudo-first-order rate constant (*k*), as follows:2
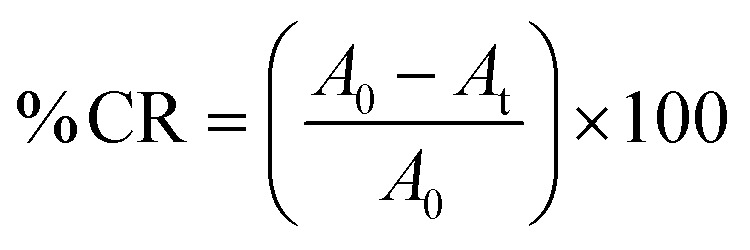
3
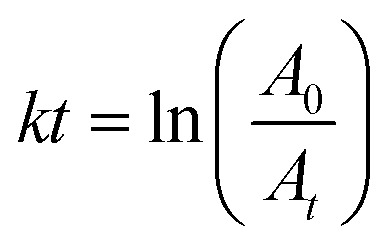
where *A*_0_ and *A*_*t*_ represent the initial absorption coefficient of pollutants and the final absorption coefficient after addition of AgNPs at time *t*, respectively. The degradation rate constant, *k*, can be determined from the slope of the line.

#### 2.4.1 Catalytic reusability experiments

The ability to reuse or recycle a catalyst is essential in determining how resilient, durable, and valuable it might be for sustainable environmental issues.^36^ This study assessed the catalytic efficacy and reusability of the synthesized AgNPs in the degradation of MB and MO dyes under UV-visible light. Suitable quantities of AgNPs and NaBH_4_ were added to a 20 mL solution of MB and MO dye for the purpose of reducing the dye pollutants. The reduction of both dyes was conducted at room temperature and neutral pH using orbital shaking for 30 and 18 minutes, respectively, until the MB and MO dye solutions became colorless. The AgNPs were isolated through centrifugation, subsequently washed with deionized water and ethanol, and then dried in an oven at 60 °C. After that, a 0.10% suspension of the dried AgNPs was prepared and used for cycle 1 in the UV cuvette. Similarly, the reusability study was repeated for the subsequent 2 cycles.^37^

### 2.5 Antimicrobial efficiency evaluation

The investigation examines the efficacy of AgNPs in inhibiting microbial proliferation by assessing their inhibitory zones against specific microbial strains to address antimicrobial resistance concerns.^38,40^ The antibacterial efficacy of the synthesized AgNPs (0.5 mM, 1 mM, and 2 mM) was evaluated by applying the agar disc diffusion method, in accordance with the Clinical Laboratory Standards Institute (CLSI, 2018) recommendations.^39^ Two Gram-positive bacteria (*S. Aureus* and *B. cereus*) and four Gram-negative bacteria (*E. coli*, *Pseudomonas* spp.,*K. Pneumoniae*, and *Salmonella* spp.) were used as test isolates. Each bacterium was inoculated on Muller-Hinton agar (MHA) plates at a concentration of 1.5 × 10^8^ colony-forming units (0.5 McFarland standard). Nanoparticles were dispersed at concentrations ranging from 0.5 to 2 mM, after which blank paper discs (OXOID) containing 20 µL of each sample were placed on the media. A broad-spectrum antibiotic, tetracycline (30 µg), served as the positive control, while autoclaved pure water functioned as the negative control. The plates were kept at 37 °C for a whole day. We used a zone-measuring ruler (Himedia, India) to measure the zone of inhibition in millimeters (mm), following the previous studies.^38–40^

## 3 Results and discussion

### 3.1 Biosynthesis of AgNPs

**Fig. 1:**
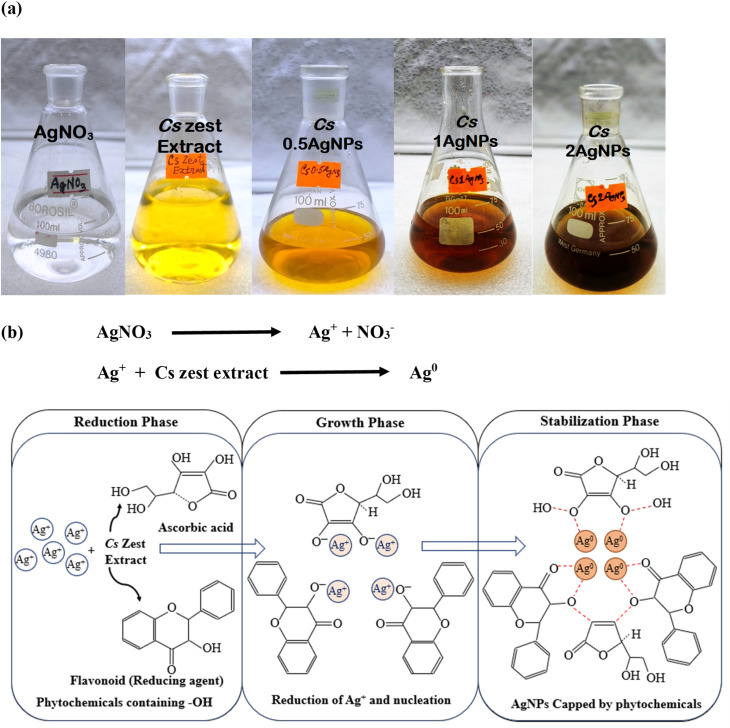
(a) Pictorial representation (left to right) of AgNO_3_ solution, *Cs* zest extract, and synthesized AgNPs of *Cs*0.5AgNPs, *Cs*1AgNPs, *Cs*2AgNPs; and (b) recommended framework for the synthesis of AgNPs from *Cs* zest extract.

The reactivity between the aqueous *Cs* zest extract and AgNO_3_ solution (precursor) was evidenced physically by the change in color from light yellow to reddish brown, which reflected the bio-reduction of Ag^+^ in the solution to Ag^0^ as displayed in Fig. 1a.^41^ The color for the three working solutions varied from light brown to wine red, depending on the concentration of silver precursors (0.5 mM, 1 mM, and 2 mM, respectively). The color change indicates regulation of AgNP size and morphology, highlighting the need to optimize the silver precursor-to-extract concentration ratio to elucidate the extract's reducing capacity for the synthesis of small, stable AgNPs. The present synthesis strategy employs the green synthesis of AgNPs using Citrus sinensis zest extract, rather than relying solely on ascorbic acid. In this approach, nanoparticle formation is governed by a complex mixture of phytochemicals rather than a single compound. The zest extract is rich in bioactive constituents, including ascorbic acid, phenolic acids (gallic, caffeic, ferulic), flavonoids (hesperidin), and terpenoids.^42^ Although a fraction of ascorbic acid undergoes thermal degradation above 70 °C, typically around 80–90 °C, yielding oxidation products such as dehydroascorbic acid, these products may still participate in redox processes during nanoparticle synthesis.^43^ More importantly, the reduction of Ag^+^ ions is primarily mediated by thermally stable phytochemicals, particularly polyphenols and flavonoids, which act as the principal reducing agents. Polysaccharides and other biomolecules further contribute to nanoparticle stabilization, as confirmed by FTIR characterization.^44,45^ Nearly all of these important phytochemicals either contain an aromatic π-system or are functionalized with heavy donor atoms, such as oxygen, nitrogen, sulfur, or chlorine, which can donate their electrons to silver ions.^42,46,47^ This confirmed the significant role of the selected Cs zest extract and the presence of preexisting phytochemicals in reducing Ag^+^ to AgNPs, leading to further synthesis and characterization. Fig. 1b displayed a recommended scheme for preparing AgNPs from *Cs* zest extract, which involved three fundamental phases. In the first phase, activation begins with the reduction of silver ions to AgNPs, which nucleate and grow. The second stage involves developing a constituent coalescence structure while reducing silver ions to increase the thermodynamic stability of the generated AgNPs. In the final phase, the AgNPs are stabilized using phytochemicals from the Cs zest extract and simultaneously serve as capping and reducing agents to prevent AgNPs aggregation.^48,49^ Furthermore, several studies have extensively described the processes underlying the reduction, nucleation, growth, and stabilization of AgNPs. Comparable investigations have reported similar growth mechanisms of AgNPs, even though the biogenic extracts employed for synthesis varied across studies.^50^ Moreover, regarding other synthesis approaches, this process is simple, quick, environmentally sound, and requires a moderate synthetic technique.

### 3.2 Optimization and characterization of AgNPs

#### 3.2.1 UV-vis spectra analysis

**Fig. 2:**
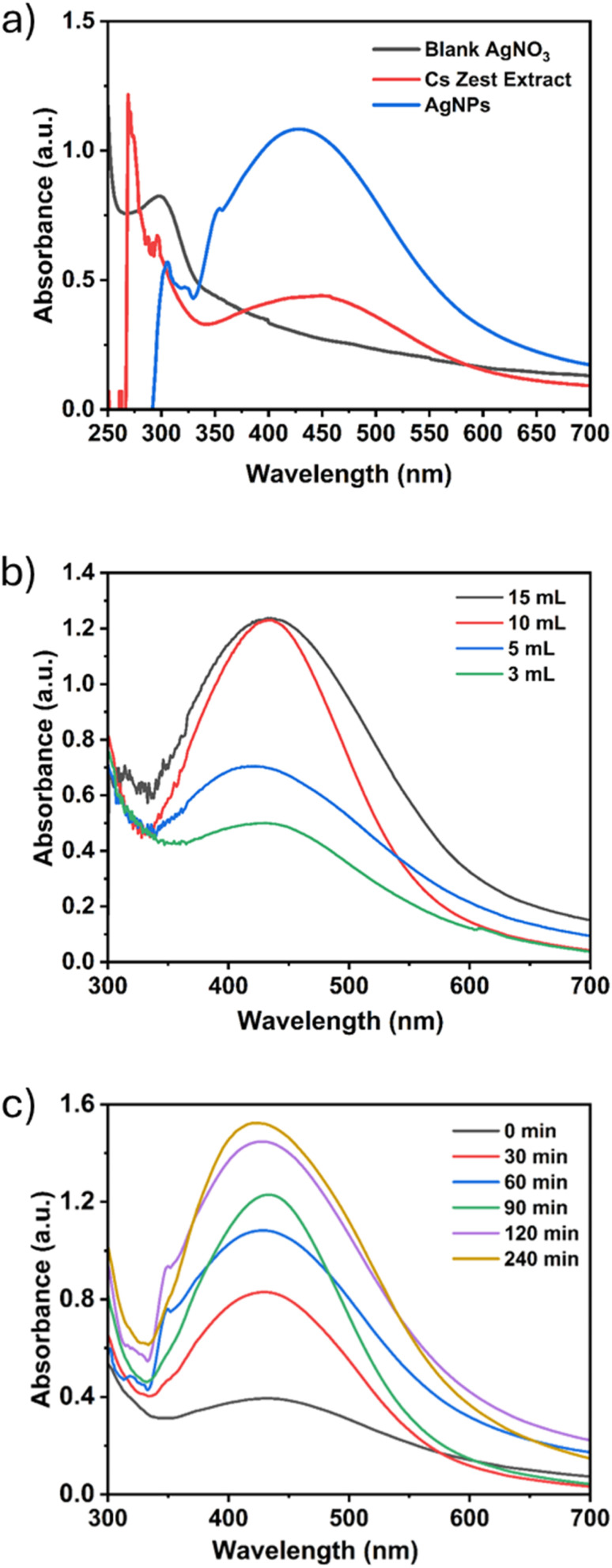
(a) UV-Vis spectra of Cs zest extract, blank AgNO_3_, and AgNPs; (b) optimization of the amount of Cs zest extract (3–15 mL) for synthesis of AgNPs; (c) optimization of the reaction time (0–240 min) for potential synthesis of AgNPs.

**Fig. 3:**
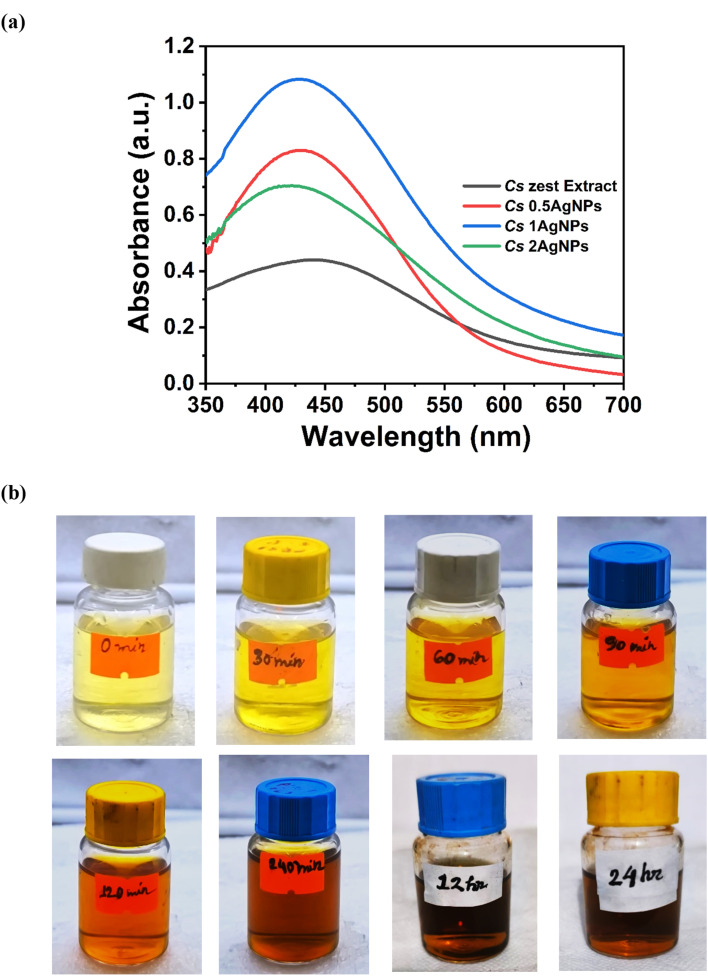
(a) UV-vis spectra of the *Cs* zest extract*, Cs*0.5AgNPs, *Cs*1AgNPs, and *Cs*2AgNPs; and (b) color change of the *Cs* 1AgNPs mixture (after 0 min, 30 min, 60 min, 90 min, 120 min, 240 min, 12 hours, and 24 hours, respectively).

The UV-Vis spectra of aqueous *Cs* zest extract and stability of synthesized AgNPs (*Cs*0.5AgNPs*, Cs*1AgNPs*, and Cs*2AgNPs) colloidal suspension were checked after 24 h incubation at different concentrations and are displayed in Fig. 3a. The optical characteristics of nanostructures are closely linked to the SPR effect, which is significantly influenced by the morphology of the nanoparticles.^51,52^ The synchronous oscillation of free electrons in AgNPs, resonating with the light wave, results in the observation of the SPR absorption band at a specific wavelength (390–450 nm) within the UV-Vis spectrum.^53^ For the zest extract, a broad absorption band was observed around 280 nm and another in the region of 450 nm, which can be attributed to the presence of phytoconstituents such as malic acid, ascorbic acid, flavonoids, polyphenols, saponins, and terpenoids.^54^ In contrast, the blank aqueous AgNO_3_ solution exhibited only a faint absorption band with *λ*_max_ near 300 nm, primarily associated with nitrate ions, and no distinct peak in the visible spectrum.^55^ Upon addition of the optimized extract to the AgNO_3_ solution, a strong SPR band appeared at 429 nm, confirming the effective formation of silver nanoparticles. The synthesis conditions of the AgNPs were optimized thoroughly. Fig. 2b depicts the influence of extract dosage on nanoparticle generation, with an increase in extract volume increasing the intensity of the SPR band to an optimal level. The SPR band became more intense and the red shift to longer wavelengths became more pronounced as the plant extract concentration increased, suggesting that the development and synthesis of AgNPs were accelerated. The optimum extract dosage was 10 mL; beyond that, no substantial increases in nanoparticle production were seen, most likely owing to active phytochemical saturation in the reduction and capping processes.^56^ The effect of reaction time on nanoparticle growth was also investigated in Fig. 2c across various time intervals (0, 30, 60, 90, 120, and 240 minutes). The gradual rise in SPR intensity over time indicates the increasing creation of AgNPs, with optimum synthesis happening at 90 minutes, after which absorption intensity stabilized, suggesting completion of the reduction process and nanoparticle development.

The UV-Vis absorption spectra of as synthesized AgNPs at different concentrations of *Cs*0.5AgNPs,*Cs*1AgNPs, and*Cs2Ag*NPs showed maximum absorption at 426 nm, 429 nm, and 424 nm, respectively, without any shift in the peak.^33^ Both *Cs* zest extract and synthesized AgNPs exhibited distinct absorption spectra, indicating their phytochemical function as capping agents during synthesis. Furthermore, an increase in AgNO_3_ concentration corresponded to a reduction in the SPR peak, a phenomenon that could be correlated with the aggregation of AgNPs.^51,52^ After 24 hours of incubation of *Cs*1AgNPs, there was no additional increase in color intensity, suggesting that all Ag^+^ had been completely nucleated and grown, as further observed in Fig. 3b.^41^ No other peaks were observed in the spectra of the three nanoparticles, confirming that the synthesized products contained only Ag. A broad, strong peak for Cs1AgNPs indicated that the nanoparticles were distributed throughout the aqueous solution, with no evidence of aggregation.

Fig. 3a: changing the precursor concentration shifts the peak position while all other synthesis parameters remain constant throughout the procedure. Therefore, as the precursor concentration decreased, the absorption maxima shifted to the left. Again, the relationship between particle size and peak position in UV-visible spectra analysis is evident.^57^ Particle size can also be estimated by UV-visible spectroscopy.^58^ Mie scattering theory can be applied to analyze optical spectra to determine the particle size of a stable suspension.^59^4
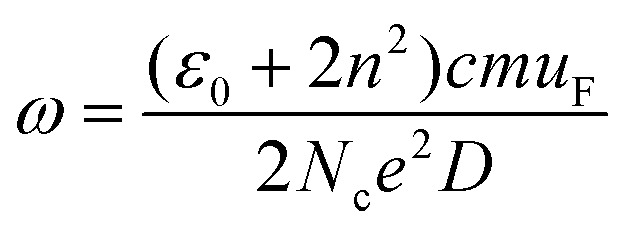
Where, *ω* denotes the full width at half maximum of a peak exhibiting a Lorentzian profile, *ε* represents the frequency-independent component of the complex dielectric constant, *n* signifies the refractive index of water, *c* indicates the speed of light, *m* refers to the mass of an electron, *u*_F_ is the electron velocity at the Fermi energy, *N*_C_ denotes the electron density per unit volume, *e* represents the charge of an electron, and *D* indicates the particle's diameter. According to this theory, particle size will be predicted as an inverse function of wavelength.^59^ Experimentally, UV-vis spectra of biosynthesized AgNPs were coherent with Mie theory. Hence, the particle sizes were 18 nm, 16 nm, and 23 nm for Cs0.5AgNPs, Cs1AgNPs, and Cs2AgNPs, respectively. The study suggests that AgNO_3_ concentration influences AgNPs production, resulting in well-defined SPR peaks and a restricted, spherical particle size distribution.^60^ The above results indicate that the 1 mM concentration of AgNO_3_ is optimal for considerable formation of *Cs*1AgNPs.

#### 3.2.2 FTIR spectra analysis

**Fig. 4:**
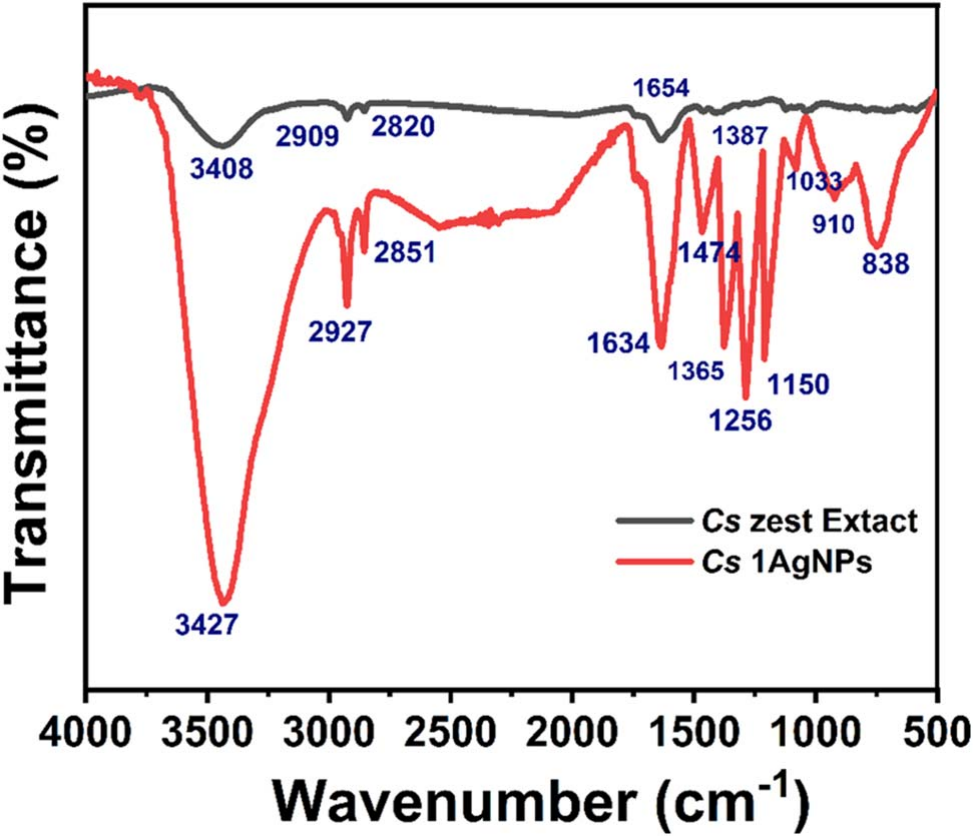
FTIR spectra of *Cs* zest *Extract, and* Cs1AgNPs.

Fig. 4 shows the FTIR analysis for both *Cs* extract and *Cs*1AgNPs, indicating the presence of significant functional groups in both samples. The figure shows that *Cs* zest extract displayed major absorption peaks at 3408, 2909, 2820, 1654, 1387, 1078, 714 cm^−1^ (Fig. 4, black). In addition, the FTIR examination of *Cs*-derived AgNPs revealed absorption peaks at 3427, 2927, 2851, 1634, 1474, 1365, 1256, 1150, 1033, 910, 838 cm^−1^, confirming the existence of biomacromolecules from *Cs* extract that would act as capping agents for stabilization of AgNPs during synthesis^61^ (Fig. 3, red). Thus, the FTIR spectrum of the Cs zest extract containing Cs1AgNPs showed significant changes in peak intensity and positions, likely owing to nanoparticle formation, capping, and stabilization.^62^ The band maximum at 3408 cm^−1^ is displaced to 3427 cm^−1^ due to O–H and N–H stretching vibrations associated with phenolic compounds derived from the *Cs* zest extract.^61^ Moreover, the frequencies at 2927 cm^−1^ and 2851 cm^−1^ correspond to aliphatic groups of C–H stretching vibrations, which are typical of triterpenoid saponins.^63^ C=C stretching (aromatic rings in hesperidin and naringin) and C=O (carbonyl in flavonoids) are also implicated by a more intense peak at 1634 cm^−1^.^63^ Additionally, the peaks at 1256 cm^−1^ and 1033 cm^−1^ indicate the presence of C–N bending vibrations in the amide group of the Cs zest extract.^64^ Furthermore, FTIR revealed that carbonyl groups from amino acid residues and proteins exhibited the highest binding capacity for metals, suggesting that proteins with amine groups could inhibit accumulation and stabilize the medium.^42,47,63^ The abundance of organic acids, phenolic compounds, antioxidants, and biomolecules such as polyphenols, terpenoids, hesperidin, polysaccharides, and steroids suggests that flavanones or terpenoids from the zest extract could be adsorbed onto the surface of AgNPs.

#### 3.2.3 XRD analysis

**Fig. 5:**
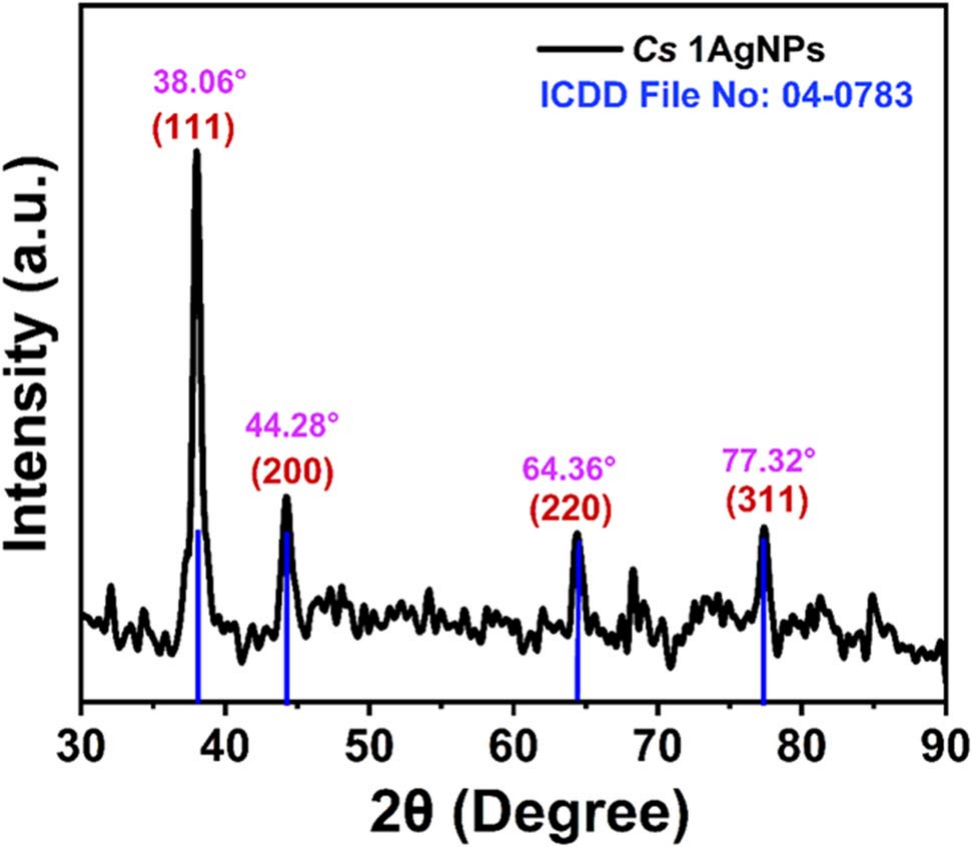
XRD analysis of *Cs*1AgNPs.

The crystalline behavior and crystallite size of the biosynthesized Cs1AgNPs were analyzed by X-ray diffraction, which provides information on the material's phase composition and structural features.^65^Fig. 5 shows the XRD patterns of Cs1AgNPs, which belong to the same space group, as indicated by Bragg's reflections. The characteristic diffraction peaks of the *Cs*1AgNPs were displayed close to 38.06°, 44.28°, 64.36°, and 77.32° in the 2*θ* range of 30°–80° in the spectrum. These peaks are assigned to the (111), (200), (220), and (311) reflection planes of the face-centered cubic (fcc) structure of Ag phases (ICDD card no. 07-0738).^66^ In Addition, residual minor spikes are observed because of the crystalline structure of the bio-organic phase on the surface of the AgNPs.^67^ However, these peaks are substantially weakened, which means that pure AgNPs are forming. It is important to mention that the relative intensity of (111) diffraction planes of *Cs*1AgNPs was higher, demonstrating AgNPs enriched in (111) facets, thus allowing more silver atoms to adhere to the (111) planes. Moreover, the evidence of additional diffraction peaks supports the participation of plant-derived chemicals as reductants, which is consistent with FTIR and UV-vis spectral analysis results.^65,66^ The average estimated crystallite dimension of *Cs*1AgNPs was 13 nm. This result closely aligns with UV-spectra analysis. As the size of the crystallite depends on the aggregation phenomena of AgNPs, AgNPs were encapsulated through bio-molecules and hence, aggregation was hindered.^68^

#### 3.2.4 SEM analysis

**Fig. 6:**
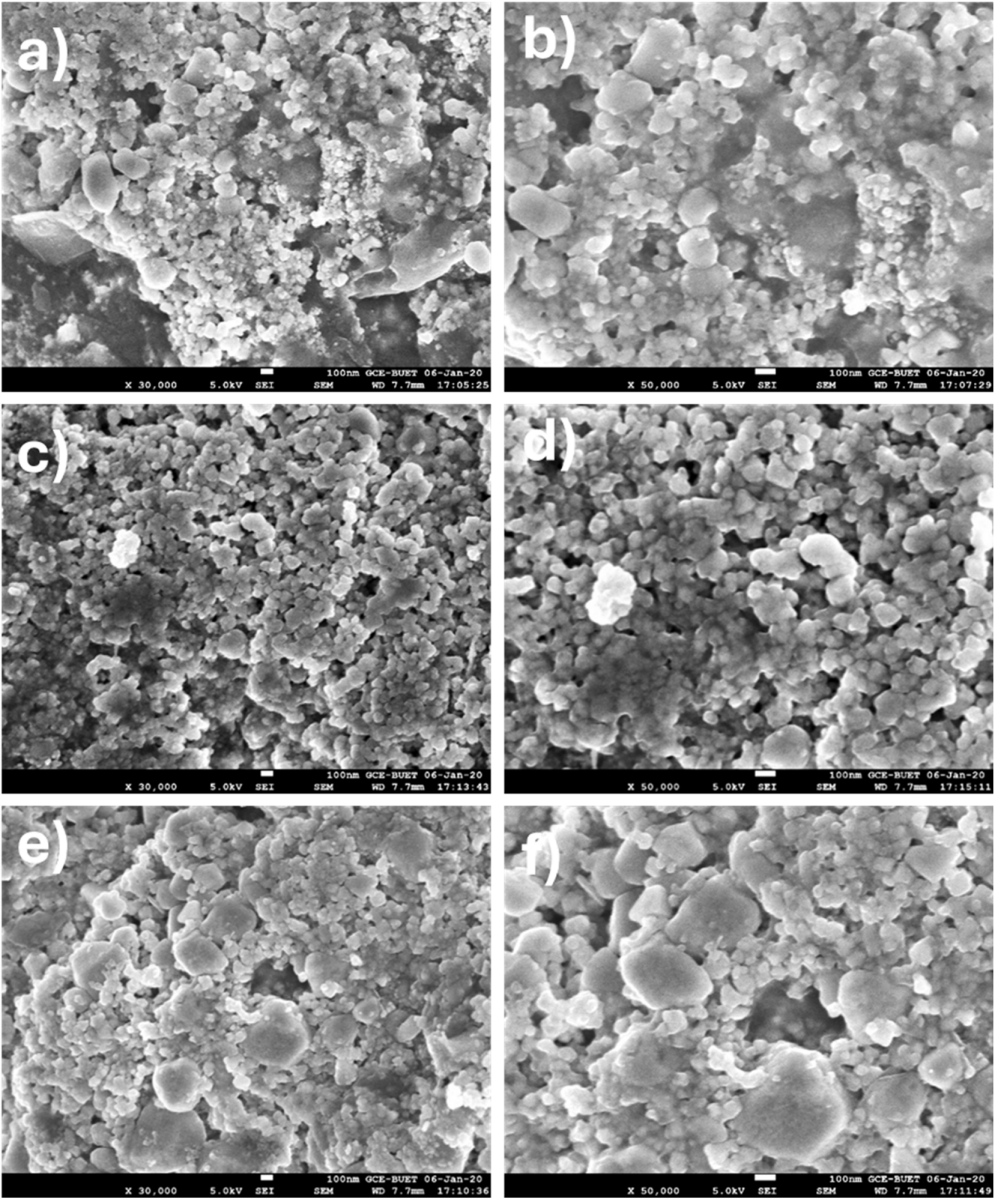
FESEM micrographs of AgNPs (*Cs*0.5AgNPs, *Cs*1AgNPs, *Cs*2AgNPs, respectively) at different magnifications: (a, c, e) 300 00× (b, d, f) 500 00×.

FESEM images were important for determining the surface morphology and texture of *Cs*0.5AgNPs, *Cs*1AgNPs, and *Cs*2AgNPs.^69^Fig. 6 shows the morphological properties of the three types of synthesized AgNPs obtained from FESEM at different magnifications. Fig. 6a, b, e, and f indicate that the *Cs*0.5AgNPs and *Cs*2AgNPs were polymorphic in form, with irregular granules, ellipsoids, rough surface morphology, and high aggregate content. They also exhibited larger particle sizes, indicating insufficient reduction and stabilization at both very low and very high precursor concentrations. At a lower concentration of 0.5 mM, the nanoparticles may exhibit lower nucleation while at higher concentration of 2 mM they provide uncontrolled growth and partial aggregation. However, Fig. 6c and d micrographs substantiate that the *Cs*1AgNPs were mostly uniformly distributed, well-dispersed, regular, and spherical in shape.^66^ They also showed relatively smaller and homogeneous nanoparticles. Some Particles were randomly granulated, rectangular, and slightly clustered, as shown in the figure. The exterior of nanoparticles is coated in an organic layer derived from phytoconstituents that function as capping agents.^36^ The enhanced structural uniformity at 1.0 mM indicates an optimal equilibrium between silver ion availability and the reduction and capping mediated by phytochemicals.^70,71^ XRD analysis revealed high-intensity crystal formations with a large crystalline area for Cs1AgNPs, which was further confirmed by SEM analysis. Crystal regions with AgNP clusters were observed on the surface of polydisperse particles, possibly due to nanoparticle aggregation triggered by solvent evaporation during sample preparation.^72^ These factors may have influenced the variance in particle size. The micrographs also showed that the particle size of Cs1AgNPs ranged from 5 nm to 30 nm, consistent with the UV-Vis spectroscopic analysis.

#### 3.2.5 TEM analysis

**Fig. 7:**
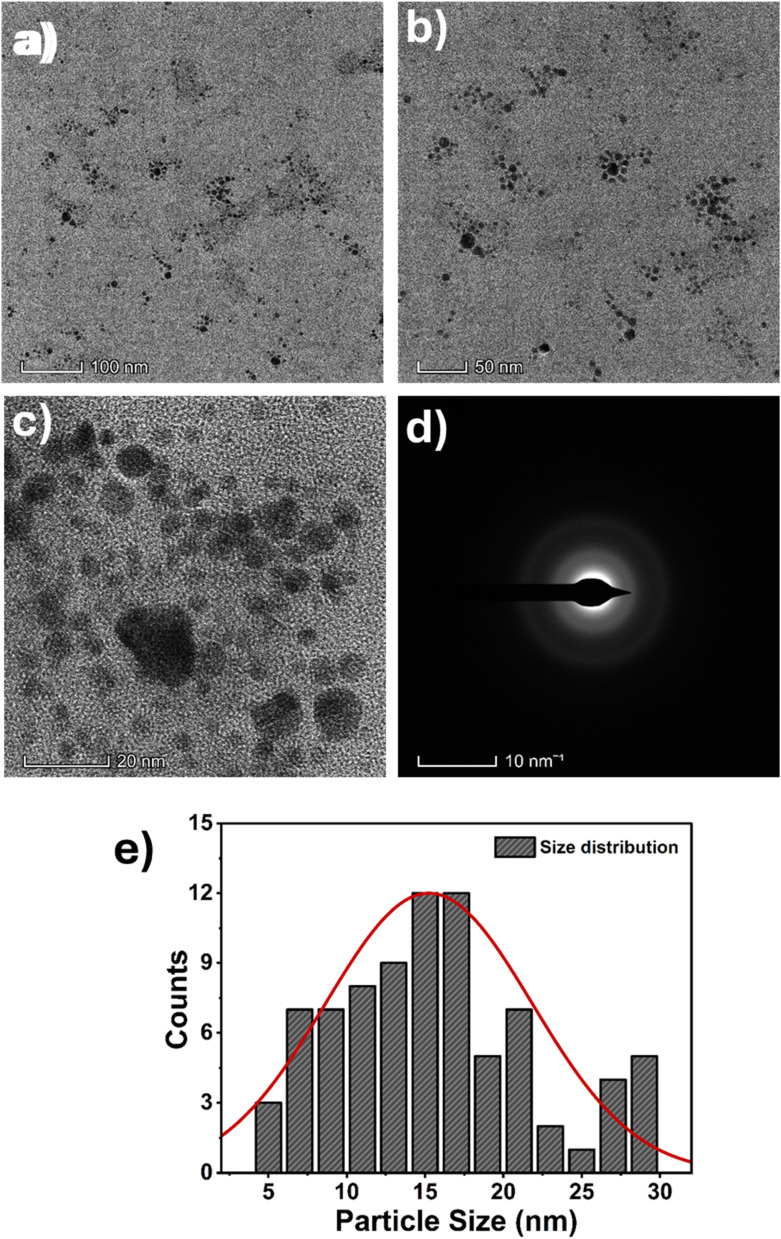
(a–c) TEM images of *Cs*1AgNPs; (d) SAED pattern of *Cs*1AgNPs; (e) particle size distribution from TEM images for *Cs*1AgNPs.

The optimized *Cs*1AgNPs synthesis was further evidenced by TEM and designed to improve the visibility of metal nanoparticles (Fig. 7). Fig. 7a–c demonstrates that polydisperse *Cs*1AgNPs display spherical and quasi-spherical morphologies. The nanoparticles exhibited transparent capped edges, which may be due to polyphenols, flavonoids, and organic amino acid groups in the Cs zest extract.^66^ The shape of the particles was mostly spherical, with some non-spherical structures observed, which is consistent with the shape of the SPR band in the UV-vis spectrum.^49,51,54^ However, Fig. 7d shows the selected-area electron diffraction (SAED) pattern, indicating the polycrystalline nature of the Cs1AgNPs,^73,74^ with indexed diffraction rings matching the fcc crystal structure of metallic silver (ICDD card no. 07-0738). These results correspond with the XRD diffractogram shown in Fig. 5. Additionally, Fig. 7e presents the particle-size distribution histogram for 100 nanoparticles, with the mean particle size determined from TEM micrographs analyzed in ImageJ. The prevailing research found that the particle size of biogenic AgNPs ranged from 5 to 30 nm. Despite this, their average particle size is 15 ± 5.3 nm, which correlates well with the estimated size from the XRD examination (13 nm) as evidenced in Fig. 7e and 5.

#### 3.2.6 EDX analysis

**Fig. 8:**
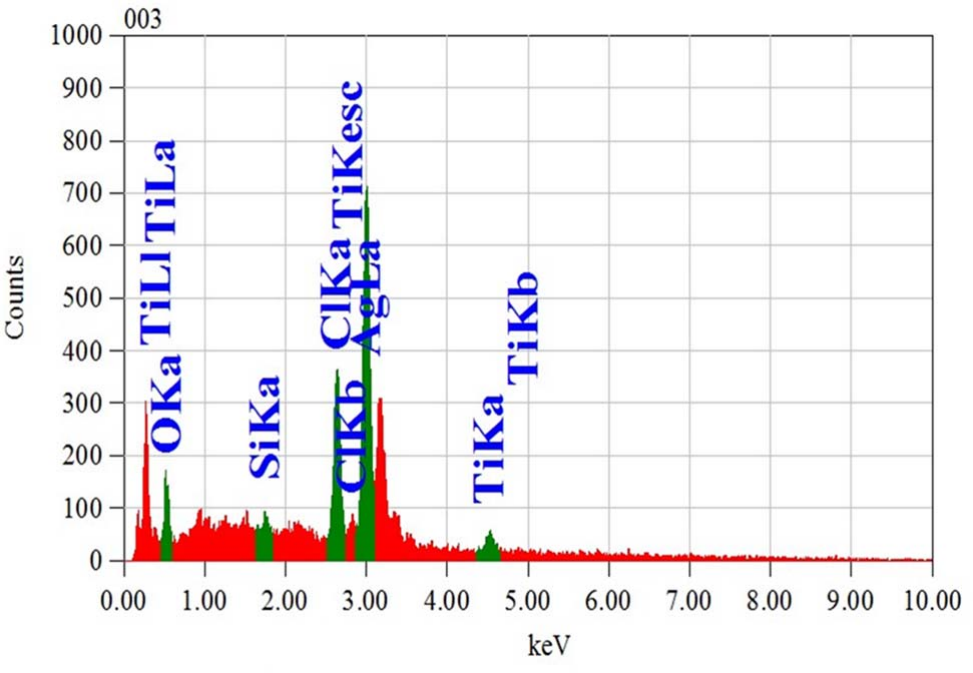
EDX spectra of *Cs*1AgNPs.

The elemental composition of the *Cs*1AgNPs was analyzed using EDX spectra from FESEM analysis (Fig. 8 and Table 1). EDX spectra provide a pronounced signal in the silver region, exhibiting significant crystalline characteristics, thus confirming the synthesis of AgNPs.^75^ Generally, metallic silver nanocrystals often exhibit an optical absorption peak at about 3 keV, attributed to surface plasmon resonance.^76^ Consequently, silver constituted the predominant element in *Cs*1AgNPs (95% Ag), as shown by the EDX profile, which exhibited a robust signal for AgNPs with lower signals from C, O, Cl, and Si peaks. Thus, better purity of bio-generated nanoparticles was discovered, with fewer C and O peaks in the EDX spectrum of *Cs*1AgNPs, indicating the existence of a biomolecule responsible for reduction and stability in the synthesized biogenic AgNPs.^65,66^ The findings were consistent with XRD and FTIR analyses, demonstrating the purity of the AgNPs.

**Table 1:** Elemental Composition of the *Cs*1AgNPs

Element	Weight %	Atomic %
O K	2.5	16
C K	1.8	6
Si K	0.2	1.5
Cl K	0.6	2
Ag L	95	74

#### 3.2.7 Catalytic degradation of MB dye

The effectiveness of biogenic *Cs*AgNPs as a catalyst was evaluated for the decomposition of MB, a non-biodegradable pollutant that is poisonous and carcinogenic, posing significant risks to aquatic environments.^77^ A UV-Vis spectrophotometer was used to monitor the absorption spectrum of MB over time, both in the presence and absence of AgNPs.

**Fig. 9:**
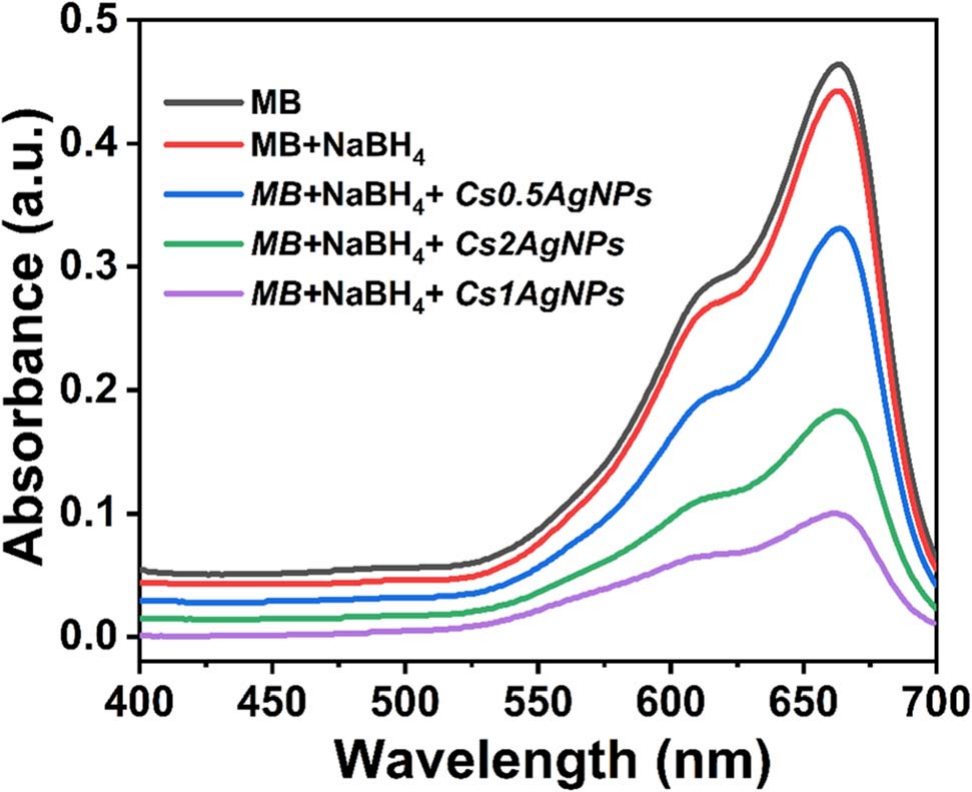
UV-vis absorption spectrum of MB dye using *Cs*0.5AgNPs, *Cs*1AgNPs, and *Cs*2AgNPs.

Nanomaterials are widely used for dye degradation due to their effectiveness in removing hazardous chemicals from industrial effluents. It is observed that micron-sized particles possess a significant surface area, which paved the way for being salient candidates for catalytic behavior.^78^ Therefore, the catalytic dye-degradation properties of Cs0.5AgNPs, Cs1AgNPs, and Cs2AgNPs were studied using MB (Fig. 9). Among the examined nanoparticles, Cs1AgNPs demonstrated the greatest catalytic performance, making them the preferred choice for future degradation research. From TEM analysis and particle size distribution discussion (Fig. 7) found that smaller and uniformly distributed nanoparticles have a higher surface area and provide more accessible active sites, which can show greater catalytic efficiency towards dye degradation. Additionally, the XRD and morphological studies confirmed that *Cs*1AgNPs had the smallest particle size distribution (15 nm) and the greatest effectiveness among the three produced nanoparticles, as shown in Fig. 9.

**Fig. 10:**
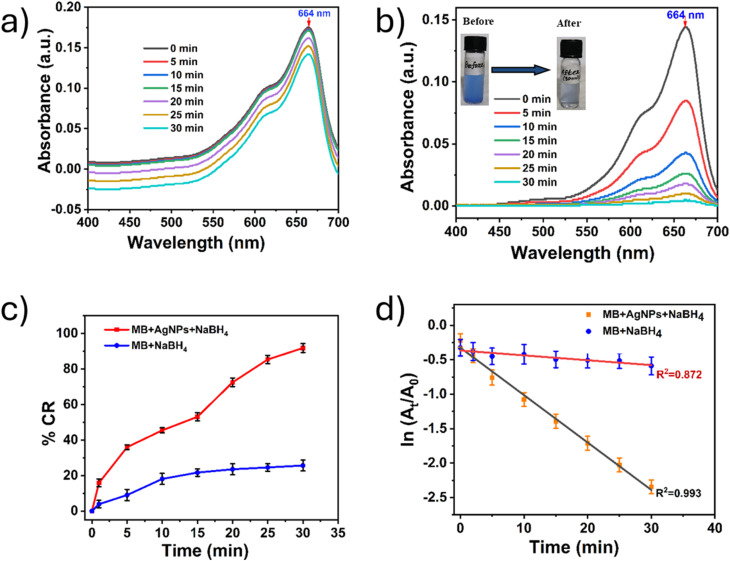
The UV-visible absorption spectrum of MB dye was analyzed over 30 minutes of catalytic reduction under the following conditions: (a) without using AgNPs, (b) using *Cs*1AgNPs, (c) the calculated % of CR of MB, and (d) the application of a pseudo-first-order kinetic model for the catalytic removal of MB.

The UV-Vis absorption of MB dye over time, both in the absence and presence of *Cs*1AgNPs, is shown in Fig. 10a and b, respectively, within the visible spectrum at 664 nm. The decomposition of MB dye was very slow when only NaBH_4_ was used as a reducing agent (Fig. 10a). Consequently, when *Cs*1AgNPs were added as the catalyst in a solution containing MB and NaBH_4,_ the degradation was achieved within 30 min. It decreased the peak intensity of UV-Vis spectra (Fig. 10b). The percentage catalytic reduction (% CR) was calculated using eqn (2), and further details are provided in Fig. 10c. Approximately 92.15% reduction was achieved with AgNPs + NaBH_4_ within 30 minutes, whereas only 19.23% was observed without AgNPs (MB + NaBH_4_). AgNPs are efficient catalytic materials owing to their higher surface area, which modifies the surface morphology, offering more active sites, leading to faster deterioration and reduction of the MB dye.^79^

To better understand the experimental findings of MB catalytic degradation, a first-order kinetic model was adopted, with the linear form outlined in eqn (3). Fig. 10d aligns well with the experimental results, with a linear correlation coefficient (*R*^2^) of 0.993, indicating that the reduction reaction follows a pseudo-first-order chemical kinetic model. Thus, the reaction rate depends on dye concentration, where higher regression values correspond to faster degradation performance.^33^ The rate constant value (*k*) for MB catalytic degradation using AgNPs was determined by applying the slope from the linear regression, in accordance with previous investigations.^80,81^ The rate constant was 0.113 min^−1^, which reflects the degradation speed, higher values for MB + AgNPs + NaBH_4_ than those observed for MB + NaBH_4_ (0.068 min^−1^).

#### 3.2.8 Catalytic degradation of MO dye

**Fig. 11:**
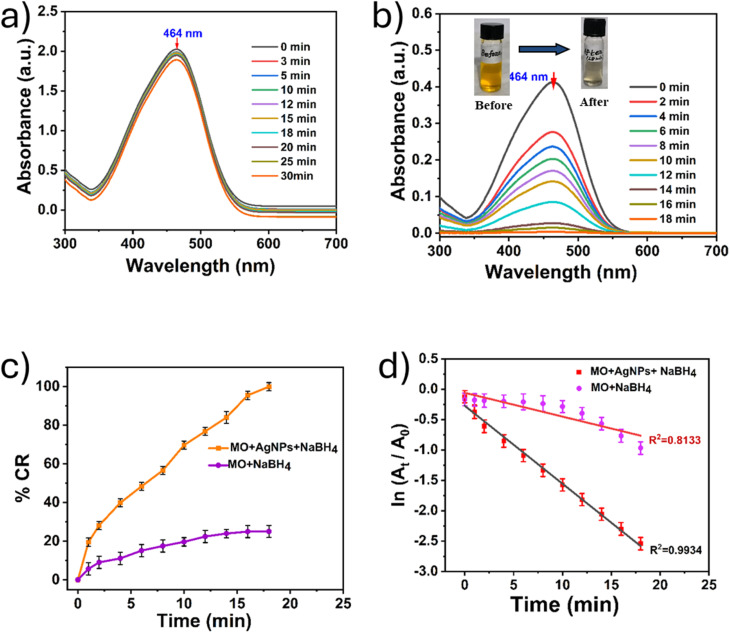
The UV-visible absorption spectrum of MO dye was analyzed over 18 minutes of catalytic reduction under the following conditions: (a) without using AgNPs, (b) using *Cs*1AgNPs, (c) the calculated % of CR of MO, and (d) the application of a first-order kinetic model for the catalytic reduction of MO.

One of the most pertinent anionic azo dyes is MO, which is widely used in textile dyeing. It shows a significant UV-Vis absorption maximum at 464 nm, which may be attributed to azo-group adsorption.^82^Fig. 11a and b show the results of research on the catalytic degradation of MO with and without AgNPs and NaBH_4_. Without the AgNPs catalyst, the breakdown of MO dye with NaBH_4_ was extremely sluggish and required a long time, as illustrated in Fig. 11a. The possibility of an extensive energy barrier between the negative ions of borohydride (BH_4_^−^) and MO may explain this.^83^ When AgNPs were added to the DI water solution of MO and NaBH_4_ the orange color of the dye disappeared within 18 minutes and the absorbance spectrum was significantly decreased (Fig. 11b). Hence the azo group (–N=N–) in MO has been altered to the colorless amine group (–NH–NH–), indicating full conversion of dye to equivalent amine molecules. Fig. 11c shows the percentage catalytic reduction (%CR) of the MO dye under different reaction conditions. The graph shows that the MO dye was slowly degraded using NaBH_4_ as a catalyst, with a degradation percentage of 18.93%. Whereas the orange line (Fig. 11c) for the (MO + AgNPs + NaBH_4_) reaction condition displayed approximately 98.93% degradation of MO accomplished in 18 min. Furthermore, Fig. 11d illustrates pseudo-first-order kinetics for the removal of MO using AgNPs + NaBH_4_. The linear curve aligns perfectly with the deterioration of MO, with an estimated constant value of 0.128 min^−1^.

**Fig. 12:**
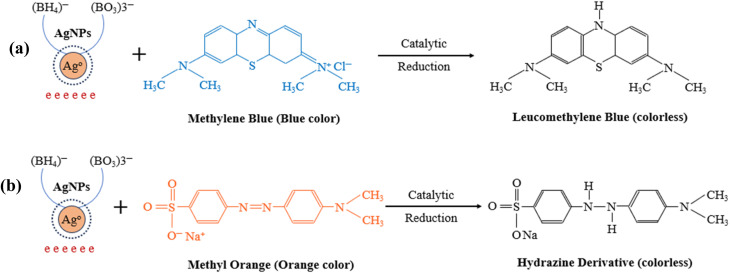
Proposed reaction mechanism for the catalytic degradation of MB and MO dye using *Cs*1AgNPs as a catalyst.

Both catalytic reduction studies (MB, MO) demonstrate that the synthesized AgNPs from the Cs zest extract degrade contaminants more effectively than other catalysts, owing to their distinctive synthesis method. Based on these findings, it was clear that the *Cs*1AgNPs acted as a significant catalyst for MB and MO, and the reduction was finished in only a few minutes. Consequently, a plausible strategy was suggested for the mitigation of these pollutants by means of electron transfer (Fig. 12). In this mechanism, NaBH_4_ functions as a nucleophile, while dye molecules (MB, MO) function as electrophiles or electron acceptors, and *Cs*1AgNPs support the electron transfer system and influence the degradation activities by acting as an electron relay system. Adsorbing both the electron donor and acceptor on the AgNPs surface *via* electrostatic interactions with the phytochemical initiates the catalytic reduction process. Subsequently, electrons are released from NaBH_4_ to the surface of the AgNPs, where they are then captured by the MB or MO dye. This interaction leads to the reduction of MB, resulting in the formation of colorless leucomethylene blue, while the decrease of MO transforms it into a hydrazine derivative. In addition, the synthesized silver nanoparticles, according to a well-documented catalytic degradation mechanism, effectively transform dye molecules into colorless and non-toxic end-products (such as CO_2_, H_2_O, and inorganic ions).^79–83^ The presence of phytochemicals from *Cs* zest extracts acts as a stabilizing and reducing agent for enhancing the efficiency of *Cs*1AgNPs catalytic degradation. Consequently, small-sized *Cs*1AgNPs with high specific surface area become effective catalysts for releasing electrons from NaBH_4_ on the AgNPs' surface. As a result, they will be effectively adsorbed by the MB and MO dyes, thereby accelerating the degradation rate.^62,78^ It is important to acknowledge that differences in experimental conditions (such as dye concentration, catalyst dosage, and degradation time) may affect the reported efficiencies. These significant influencing parameters have been widely studied in earlier works, mostly for synthetic dye systems.^32,84,85^ To properly assess its practical implementation under typical environmental conditions, however, future research should examine the effectiveness of a developed system in more complex real-world wastewater matrices.

#### 3.2.9 Reusability of AgNPs as a catalyst

**Fig. 13:**
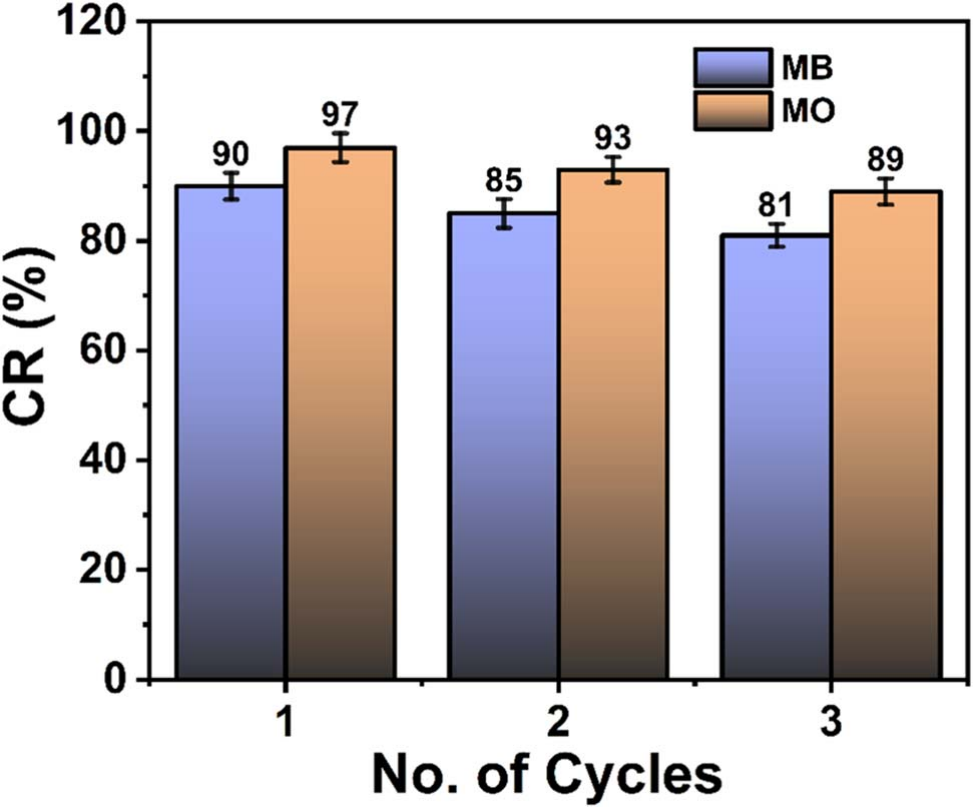
Reusability investigation of *Cs*1AgNPs for the reduction of MB and MO dyes.

The reusability of the *Cs*1AgNPs catalyst was assessed (Fig. 13) *via* three catalytic cycles to evaluate its operational stability and economic viability for dye removal, and also for industrial applications.^86^ The recycling studies demonstrated that *Cs*1AgNPs retained exceptional catalytic activity after multiple uses, with only a modest decrease in efficiency. The degradation efficiency of MB was 90% after the first cycle and reduced to around 85% after the third cycle, demonstrating robust structural integrity and enduring catalytic activity of the nanoparticle. Similarly, MO exhibited degradation efficiencies of 97%, 93%, and 89% in subsequent cycles, demonstrating the resilience of *Cs*1AgNPs in repeated catalytic applications. The modest decrease in effectiveness after three cycles may be ascribed to partial surface fouling of active sites, adsorption of intermediate by-products, or minor aggregation of AgNPs during recovery and reuse.^87^ Nonetheless, the overall performance demonstrates that *Cs*1AgNPs can be recycled efficiently using simple washing and drying procedures, with minimal loss of efficacy, underscoring their promise as a reliable and economical catalyst for practical wastewater treatment applications.

### 3.3 Antibacterial activity assessment

**Fig. 14:**
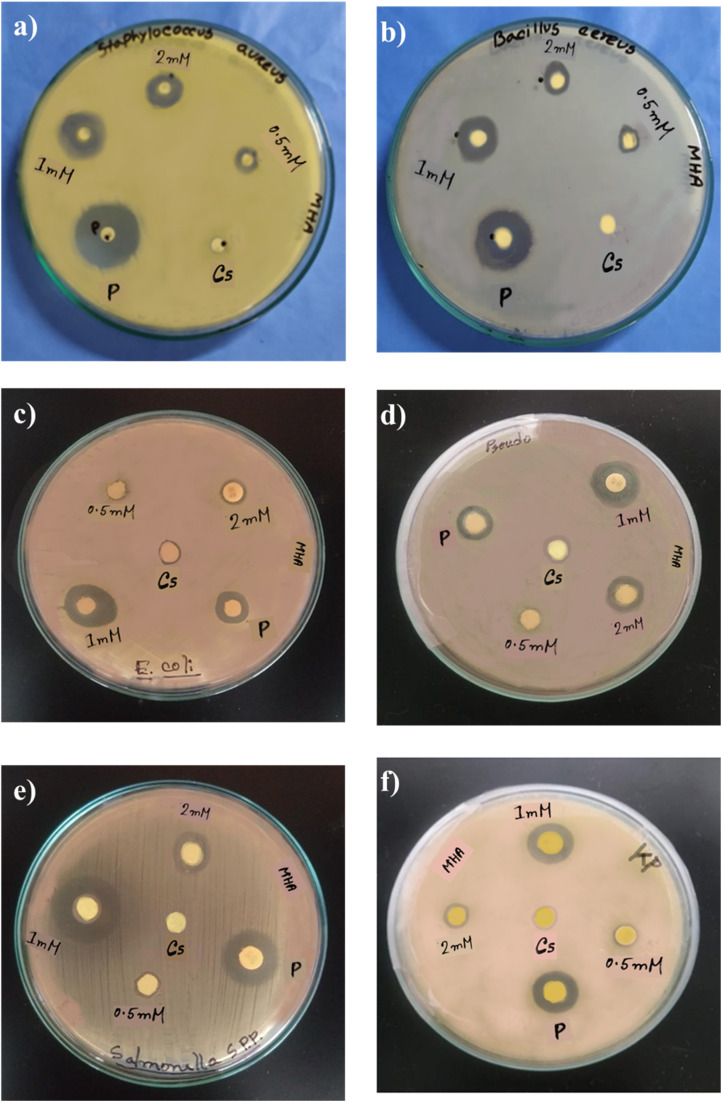
Zone of inhibition of *Cs*0.5AgNPs, *Cs*1AgNPs, and *Cs*2AgNPs for *Bacillus cereus*,*Staphylococcus aureus*,*Escherichia coli*,*Pseudomonas species*,*Salmonella species*, and *Klebsiella pneumoniae*.

The antimicrobial efficacy of the liquid zest extract of *Cs* and the AgNPs produced from it was analyzed against six bacterial strains, including *Staphylococcus aureus*,*Bacillus cereus*, *Escherichia coli*, *Pseudomonas species*, *Salmonella species*, and *Klebsiella pneumoniae* (Table 2 and Fig. 14). A prominent zone of inhibition was observed at 20 µg mL^−1^ concentration for each type of biosynthesized AgNPs, which showed promising activity when compared to the fruit zest extract.

**Table 2:** Zone of Inhibition (mm) for *Cs*0.5AgNPs*, Cs*1AgNPs, and*Cs*2AgNPs tested against different Gram-positive and Gram-negative bacteria

Bacterial strains	AgNPs at different concentrations (mM)	Zone of inhibition (mm)	*Cs* zest extract (mm)	Positive control^a^ (mm)
*Staphylococcus aureus*	*Cs*0.5AgNPs	2 ± 0.26	—	12.0 ± 0.7
	*Cs*1AgNPs	9 ± 0.19		
	*Cs*2AgNPs	7 ± 0.16		
*Bacillus cereus*	*Cs*0.5AgNPs	2 ± 0.32	—	11.0 ± 0.16
	*Cs*1AgNPs	8 ± 0.23		
	*Cs*2AgNPs	5 ± 0.19		
*Escherichia coli*	*Cs*0.5AgNPs	1 ± 0.42	1 ± 0.01	13 ± 0.17
	*Cs*1AgNPs	14 ± 0.22		
	*Cs*2AgNPs	8 ± 0.18		
*Pseudomonas species*	*Cs*0.5AgNPs	2 ± 0.24	1 ± 0.11	14 ± 0.35
	*Cs*1AgNPs	17 ± 0.1		
	*Cs*2AgNPs	10 ± 0.31		
*Salmonella species*	*Cs*0.5AgNPs	9 ± 0.19	—	19.0 ± 0.16
	*Cs*1AgNPs	23 ± 0.24		
	*Cs*2AgNPs	13 ± 0.13		
*Klebsiella pneumoniae*	*Cs*0.5AgNPs	7 ± 0.11	1 ± 0.02	12 ± 0.21
	*Cs*1AgNPs	15 ± 0.15		
	*Cs*2AgNPs	8 ± 0.25		

a Tetracycline was used as positive control.

*E. coli*, *P. species*, *S. species*, and *K. pneumonia* (Gram-negative) exhibited the largest zone of inhibition of 14 ± 0.22, 17 ± 0.1, 23.0 ± 0.24, and 15 ± 0.15 mm, respectively, in *Cs*1AgNPs in comparison to the other two types of nanoparticles. Additionally, two Gram-positive bacterial strains, *B. cereus* and *S. aureus*, showed the smallest inhibition zones of 8 ± 0.23 mm and 9 ± 0.19 mm, respectively, with *Cs*1AgNPs. Meanwhile, DI water served as a negative control for the solvent system. It had no inhibitory effect on any of the selected bacterial species. Fig. 13 also indicated that the *Cs* zest extract exhibited very slight and sometimes didn't show any antibacterial action against both the designated Gram-positive and Gram-negative bacteria, demonstrating that the biosynthesized AgNPs are significantly responsible for this antibacterial impact. However, with increasing AgNP concentration, antibacterial activity was observed (Fig. 14). These observations indicated that Cs0.5AgNPs, Cs1AgNPs, and Cs2AgNPs exhibited comparable antibacterial activity against both Gram-positive and Gram-negative bacteria. Among the three types, *Cs*1AgNPs, with the smallest particle size, exhibited the greatest region of restriction. The antibacterial efficacy of AgNPs is affected by their diminutive particle sizes and extensive surface area, facilitating their adhesion to microbial cell wall surfaces.^66,78,79^

**Fig. 15:**
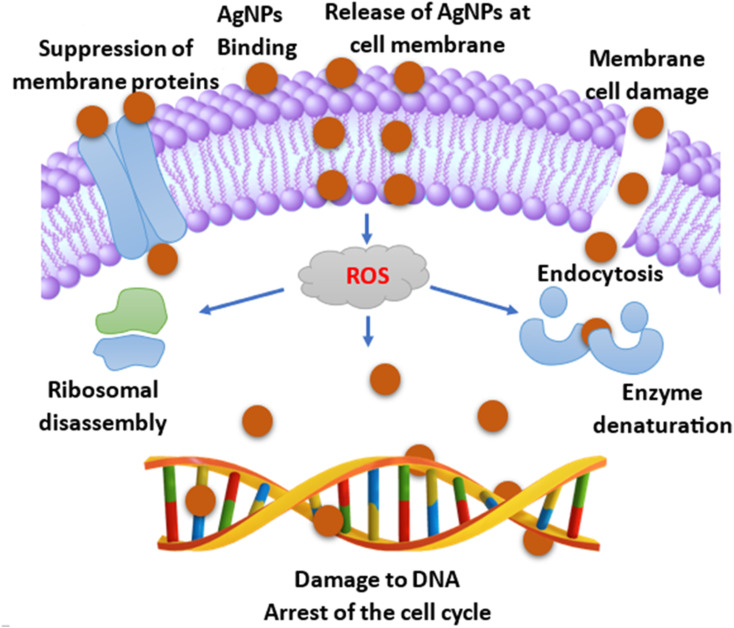
Probable antimicrobial mechanism of *Cs*1AgNPs.

Moreover, a reduced region of resistance has been seen for Gram-positive bacteria compared to Gram-negative bacteria. Gram-positive bacteria possess thicker, stiffer coatings of peptidoglycan that inhibit the penetration of AgNPs into the cell wall.^88,89^ However, surface encapsulation of *Citrus sinensis* zest extract might also contribute to their enhanced antibacterial activities.^63,64^*Salmonella species* was mostly affected. *Bacillus cereus* was least affected by all three types of nanoparticle samples and the positive control, indicating the highest resistance. The biological proteins are become inactive when exposed to synthetic nanoparticles; however, the precise mechanisms by which AgNPs inhibit bacterial growth are unclear.^90^ Furthermore, the antibacterial efficacy of AgNPs is generally attributed to their strong interaction with the bacterial cell membrane, which subsequently leads to intracellular damage. Upon exposure, AgNPs electrostatically adhere to the negatively charged bacterial cell surfaces, causing membrane deformation, altered permeability, and disruption of integrity, resulting in the leakage of essential intracellular components.^91,92^ The mechanism behind the antimicrobial action of *Cs*1AgNPs was illustrated in Fig. 15. The AgNPs progressively release Ag^+^ ions, which may infect bacterial cells and engage with thiol-containing enzymes, ribosomal subunits, and nucleic acids, leading to enzyme inactivation, obstruction of protein synthesis, and repression of DNA replication.^91,93^ Moreover, AgNPs facilitate the production of reactive oxygen species (ROS), such as superoxide radicals and hydrogen peroxide, which elicit oxidative stress and compromise cellular biomolecules. The synergistic effects of membrane rupture, intracellular silver ion toxicity, and ROS-mediated oxidative damage eventually impede bacterial growth and cause cell death. Different pathways for AgNPs action were suggested.^94–98^ The pathway can be with the cell envelope (*e.g.*, membrane, peptidoglycan), significant structural molecules (*e.g.*, proteins, nucleic acids), or in biochemical pathways. Antibacterial investigations suggest biosynthesized AgNPs could be used in pharmaceutical applications.

## 4 Conclusions

Here, a low-cost, environmentally friendly, and straightforward bio-reduction method for producing AgNPs in aqueous medium using aqueous *Cs* zest extract, demonstrating a promising and long-term strategy for biological generation of multifunctional nanomaterials. The phytochemicals in Cs zest extract, including organic acids, flavonoids, phenolics, and terpenoids, function as natural reducing agents, stabilizers, and antioxidants. This enables the environmentally friendly production of AgNPs, free from hazardous chemicals and high-energy processes. The chemical nature and morphology of the as-prepared AgNPs have been determined by using a variety of analytical methods, including UV-Vis, FTIR, XRD, SEM, TEM, and EDX. The findings confirm that the synthesized AgNPs are encapsulated with phytochemicals from Sl extract, as seen by the color change and UV-vis absorption, suggesting the reduction of Ag^+^ to Ag^0^. The majority of functional groups were detected *via* FTIR, which corresponds to the total phenolic compound analysis. The produced AgNPs exhibit a spherical morphology with a particle size distribution in the 5–20 nm range, as confirmed by XRD, SEM, and TEM examination. This study employed the generated AgNPs as a catalyst to degrade MB and MO dyes, using NaBH_4_ as the reducing agent. An aqueous solution of *Cs*1AgNPs can catalytically diminish MO and MB by approximately 98.93% and 92.15%, respectively, after 18 and 30 minutes. The catalytic reduction reactions followed a first-order kinetic model. The obtained kinetic constants were 0.128 min^−1^ for MO and 0.113 min^−1^ for MB, indicating efficient and consistent reaction behavior. Furthermore, the antibacterial efficacy of the three types of synthesized AgNPs was assessed against six selected bacterial strains, comprising both Gram-positive and Gram-negative bacteria. *Cs*1AgNPs demonstrated excellent antibacterial activity, with particularly high efficacy against Gram-negative pathogens than against Gram-positive ones. The dual functionality of these green-synthesized AgNPs makes them ideal for both integrated environmental remediation and biomedical applications, enabling safer nanomaterial development through sustainable approaches. Considering the promising outcomes in comparison with prior research, future investigations should focus on evaluating performance within complex wastewater matrices and exploring mechanistic insights to enhance scalability and practical applicability.

## Conflicts of interest

There are no conflicts of interest to declare.

## Data Availability

All data supporting the findings of this study are available within the article.
